# PUCHIK: A Python Package To Analyze Molecular Dynamics Simulations of Aspherical Nanoparticles

**DOI:** 10.1021/acs.jcim.4c02128

**Published:** 2025-02-10

**Authors:** Hrachya Ishkhanyan, Alejandro Santana-Bonilla, Christian D. Lorenz

**Affiliations:** † Institute for Informatics and Automation Problems of the National Academy of Sciences of the Republic of Armenia, 0014 Yerevan, Republic of Armenia; ‡ Department of Physics, King’s College London, London WC2R 2LS, United Kingdom; ¶ Department of Engineering, King’s College London, London WC2R 2LS, United Kingdom

## Abstract

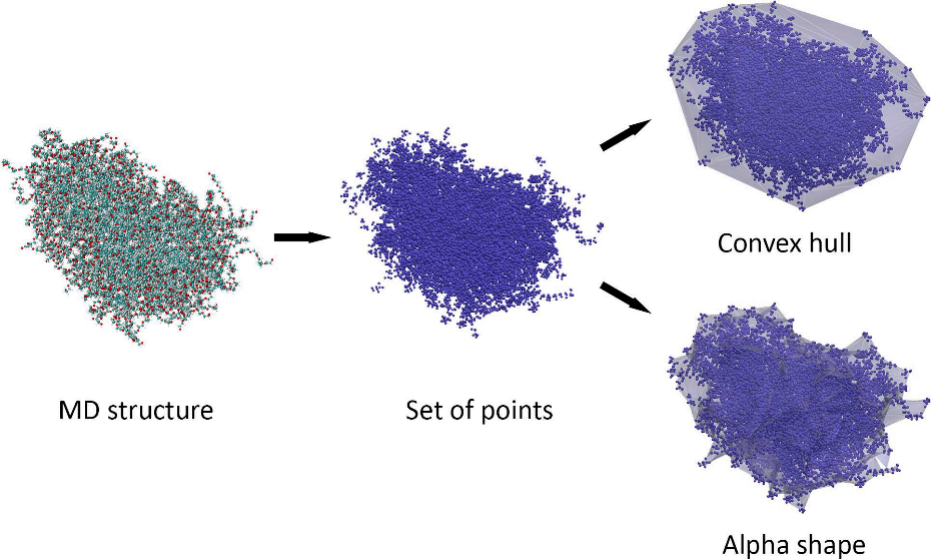

Accurately describing a nanoparticle’s interface is crucial for understanding its internal structure, interfacial properties, and ultimately, its functionality. While current computational methods provide reasonable descriptions for spherical and quasi-spherical nanoparticles, there remains a need for effective models for aspherical structures such as capsules and rod-like systems. This work introduces Python Utility for Characterizing Heterogeneous Interfaces and Kinetics (PUCHIK), a novel algorithm developed to describe both spherelike and aspherical nanoparticles. With an accurate description of the location of the interface of the nanoparticle, this algorithm then allows for various other important quantities (e.g., densities of different atom/molecule types relative to the interface, volume of the nanoparticle, amount of solubilized molecules within the nanoparticle) to be calculated. Our software development, we focused on providing good performance to computationally demanding projects, while ensuring that the methodological approach can be adapted as a protocol for other code implementations.

## Introduction

Nanoparticles have promising applications in biomedicine, including targeted drug delivery,^1−5^ medical imaging, regenerative medicine, and biosensing.^6−10^ Understanding nanoparticle interfacial properties is critical as these properties dictate interactions with solutes, substrates, and other particles. Modifying the surface,^11,12^ shape, size, and surface area of nanoparticles allows for functional optimization tailored to specific applications.^13^

Molecular dynamics (MD) simulations are a powerful tool for obtaining atomistic detailed information on the underlying physics of various systems, including liquid–nanoparticle interfaces.^14−19^ Analysis of these simulations enables the quantification of useful properties such as volume, hydration, density profiles, solubilization of different molecules inside of the nanoparticle, etc. There are several methods to describe surfaces and interfaces, such as the marching cubes algorithm,^20^ Willard-Chandler method,^21^ the intrinsic core–shell interface (ICSI) method^22^ and the cone algorithm.^23^

While the majority of these methods provide good results when constructing interfaces for symmetric nanoparticles, they are generally less effective for aspherical nanoparticles. The capability of providing an accurate description of the interface of nanoparticles independent of their shape is particularly important, because surface effects are dominant in their functionality. MD simulations are increasingly used to understand aspherical nanoparticles (e.g., wormlike micelles)^24−27^ and how the morphology of soft nanoparticles change under different conditions.^28−30^ In these studies, the interface is characterized either by investigating a given axis of the nanoparticle or by using radial distributions for atoms thought to be at the interface to identify their local environment.

This work proposes an algorithm capable of defining core–shell interfaces for both symmetric and asymmetric nanoparticles composed of any type of material. It also includes volume and density calculations relative to the interface along with a detailed Python implementation that addresses computational demands. This approach aims to serve as a guide for optimizing other computational codes.

Determining the water–core interface of a nanoparticle involves identifying the shape formed by the atoms in the hydrophobic core, similar to a computational geometry problem of computing the shape of a finite set of points. Edelsbrunner et al.^31^ introduced alpha shapes, which generalize the convex hull to better capture irregular geometries. In this work, interfaces are initially constructed using convex hulls but can be switched to alpha shapes for improved accuracy. The convex hull approach is faster, while alpha shapes are more effective for handling concave, bent, or irregular structures. The structural details provided by the alpha shape method allows for description of solid, hollow, and mesoporous materials.

## Methods

Python Utility for Characterizing Heterogeneous Interfaces and Kinetic (PUCHIK = which means “balloon” in Armenian) is primarily built on three key Python packages: SciPy,^32^ MDAnalysis,^33,34^ and Cython.^35^ SciPy is utilized for constructing the interface through its ConvexHull class, which acts as a Python wrapper for the Qhull library.^36^ MDAnalysis facilitates the reading of trajectories and topologies and provides a simple selection language resembling those used in CHARMM^37^ and VMD.^38^ The computationally intensive portions of the code are optimized using Cython, resulting in significant performance improvements. A more thorough discussion of optimizing code performance is provided in the Electronic Supporting Information (ESI). Finally, alpha shapes are implemented in C++ using the computational geometry algorithms library (CGAL).^39^

### Implementation

The calculations using PUCHIK are done in four steps: convex hull construction, discretization of the simulation box, calculation of distances from each cell to the interface, and calculation of densities in each cell of the discretized box. As mentioned above, PUCHIK uses a convex hull to describe the interface between the nanoparticle and the rest of the solute. A convex hull is defined as the intersection of each and every convex set containing the points in question and, hence, the smallest convex set for the set of points. To define the desired set of atom coordinates, upon which the hull will be constructed, an instance of the *Interface* class is created, which requires the directory path(s) to the topology and trajectory files. MDAnalysis readers are used to read the trajectory, and therefore a large variety of different formats of trajectory files can be analyzed, e.g., CHARMM, AMBER,^40^ and GROMACS.^41,42^ After loading the trajectory, the user can utilize the selection language of MDAnalysis to identify the atoms that make up the main structure. For example, the atoms comprising the hydrophobic core of a micelle can be selected. The second step divides the simulation box into a grid with *nn* cubic subdivisions (“cells”), where *n* is inputted by the user. Then, the distances between the centers of each cell and the convex hull are calculated. For each cell center, a check is performed to determine whether it is inside or outside the convex hull. If the cell center is inside the convex hull, then the distance is negative. Finally, the densities of the molecules in question are calculated and normalized for each cell.

PUCHIK can be configured to use an alpha shape^31^ rather than a convex hull to construct the interface. An alpha shape is a generalization of the convex hull that captures the shape of a set of points by ”scooping out” empty spaces between them. It uses a parameter - α, which represents a radius of a circle that is rolled over the point cloud, removing regions that do not fit within the boundary defined by the points. Smaller alpha values provide a more-detailed representation of the interface, while as α → *∞*, the interface constructed using an alpha shape becomes the same as that constructed using the convex hull. In PUCHIK, alpha is a free parameter. If it is not provided by the user, an optimal alpha will be chosen as described in the CGAL library documentation. Alpha shapes provide a more-concave representation of the interface, enabling more precise definitions at the cost of a slight decrease in computational performance. Both methods provide the atom IDs for vertices, faces, and edges of the interface, enabling the construction of a detailed mesh representation of the nanoparticle.

In its current version, PUCHIK also supports the calculation of solubilized molecules inside the convex hull. This can be used to calculate the number of trapped water molecules and encapsulated small molecules, etc. A SciPy convex hull object also contains information about its volume and surface area. With the *Interface* objects *calculate_volume* method, these data are easily retrieved.

### Package Structure and Usage

PUCHIK is designed for ease of use and efficiency. Users can accomplish tasks such as density calculations with minimal coding, often within as few as three lines. PUCHIK is executed as a script from within a Python interpreter and currently does not support execution from a command line. At the time of the writing, the package consists of two subpackages: *core* and *utilities*. The *core* package contains the main class that is going to be used, called **Interface**, while the *utilities* package contains a few useful tools that will be described later in this section. To create an **Interface** object, the user should pass the directory paths to the topology and trajectory files. As discussed previously, the default way of determining the interface of the aggregate to be studied with PUCHIK is using a convex hull, but if the users would prefer to use an alpha shape, this can be inputted using the **use_alpha_shapes** logical.

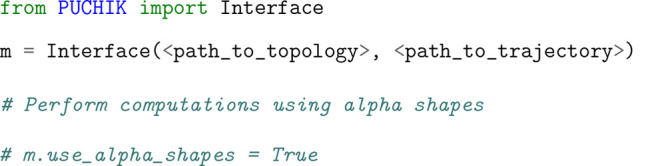



Since MDAnalysis is used to read all of the files from the molecular dynamics (MD) simulations, PUCHIK supports every file format that MDAnalysis supports. Then, the user will need to specify the atoms that make up the structure of interest, e.g., the core of the nanoparticle, with **select_structure**:

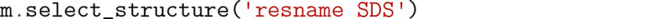



Note that atom selection is the same as that used within MDAnalysis. Finally, the **calculate_density** method can be called to calculate the density of the desired molecule type as a function of distance from the interface. The user will utilize MDAnalysis selections to identify the atoms that will be used within the density calculation:

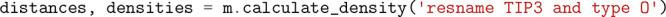



The above code snippet calculates the density of the O atoms in the water molecules, as a function of distance to the interface of the selected structure. The volume of the structure can be obtained by the **calculate_volume** method. Surface area can also be returned from the same method by setting the keyword argument *area* to **True**.

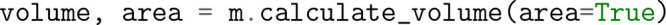



Both the **calculate_density** and **calculate_volume** methods can be customized by changing default parameters. The *start*, *skip*, and *end* parameters allow the user to identify the portion of the trajectory that the analysis is performed over. *norm_bin_count* specifies the number of divisions within the simulation box required for the density normalization process. This can be tweaked, depending on the size of the system, to achieve more-precise calculations. Dividing the MD simulation box into 10 Å segments typically yields the optimal results. For example, setting the *norm_bin_count* parameter to 10 or 12 works well when the box dimensions are 10 nm × 10 nm × 10 nm or 12 nm × 12 nm × 12 nm, respectively. Finally, since density calculations are parallelized across every CPU core by default, the user has an option to turn multiprocessing off by setting the parameter *mp* to **False**, or to reduce the number of cores utilized by the code by setting *cpu_count* to the desired number.

In simulated systems that contain multiple aggregates of molecules, PUCHIK can be used to quickly identify and analyze each aggregate separately. The *utilities* package provides the user with extra functionality to find clusters of molecules and center and make the nanoparticle whole if it crossed the simulation box. Clusters can be found by instantiating the **ClusterSearch** class, selecting the atoms to be clustered and running the **find_clusters** method:

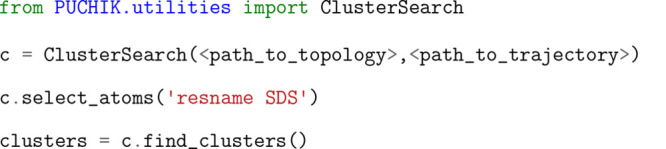



The **find_clusters** method returns a list of lists each containing residue ids for a separate cluster. A typical procedure for calculating densities in a simulation with multiple nanoparticles is as follows:

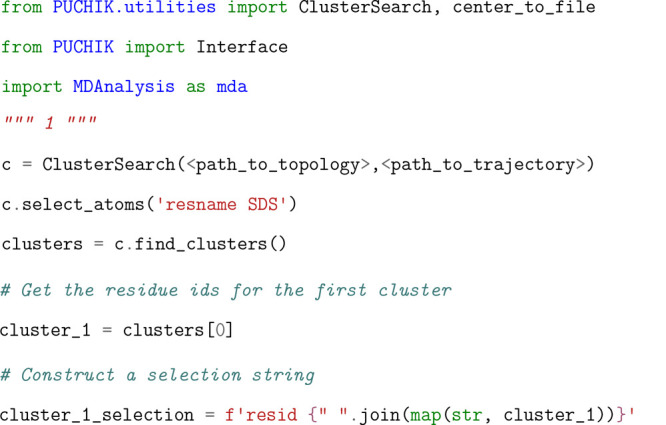

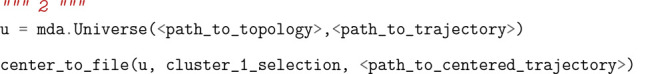

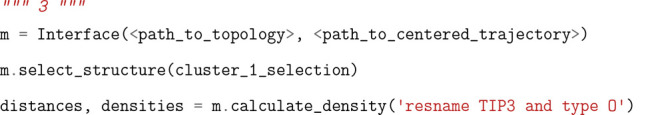







In the first part of the code, cluster IDs are obtained, and the first cluster is saved in the **cluster_1_selection** variable. The cluster is then made whole, centered within the box, and saved in a separate trajectory file by using the **center_to_file** function. Alternatively, the **center_in_memory** function can be used to achieve the same result directly on an MDAnalysis **Universe** object in-place. In the final step, the new trajectory is loaded, the desired cluster is selected as the main structure, and density calculations are performed. This routine ensures the absence of PBC-related artifacts and provides accurate density estimations.

PUCHIK is developed to be modular, and each of its functionality is independent. This is done to make it extendable and ease future development and implementation of custom user-defined functions.

## Results

### Density Calculations

**Figure 1:**
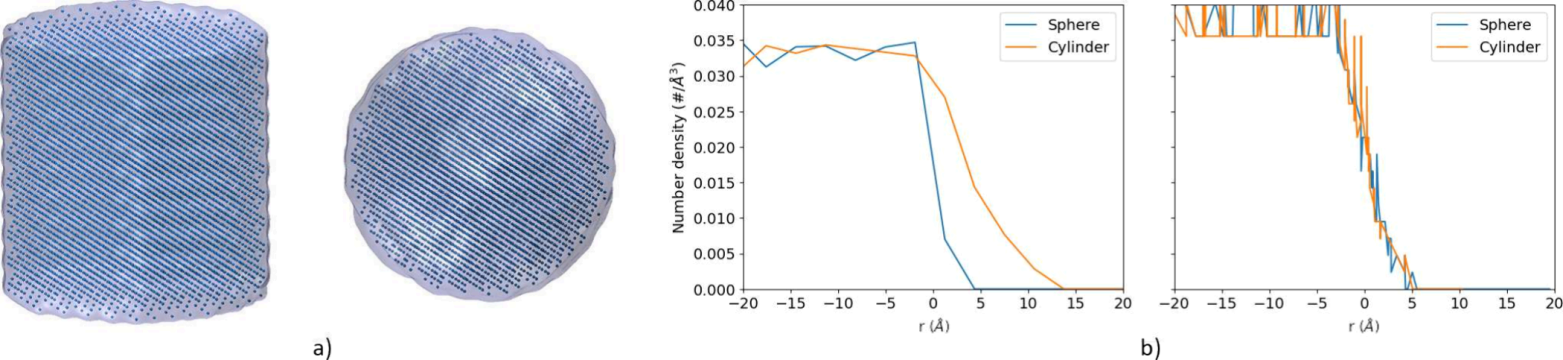
(a) Test structures with known density and volume: cylinder (left) and sphere (right). (b) Their corresponding number of atoms per volume as a function of the distance from the interface (negative values are inside the core of the nanoparticle) estimated by a standard approach (left) and the PUCHIK algorithm (right).

**Figure 2:**
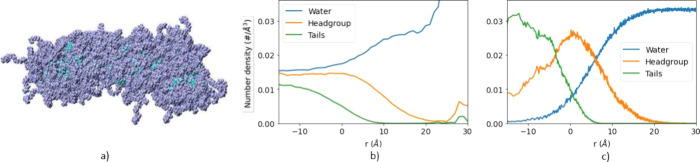
(a) Snapshot of an elongated TX100 micelle. Number of atoms per volume of water, hydrophobic tail, and hydrophilic head of Triton X-100 surfactant, as a function of distance from the interface of the hydrophobic core of the micelle. Comparison of results from two algorithms: (b) nanoCISC and (c) PUCHIK.

Two model atomic assemblies, a cylinder with a radius and half-height of 2.9 nm, and a sphere with a radius of 2.9 nm (Figure 1a), are used to evaluate the accuracy of the densities calculated using the methods within this software. The number density of the particles within the bulk of each assembly can be estimated to be 0.0375 Å^–3^. The *norm_bin_count* was set to 8 for this specific calculation. In Figure 1b, the number density of particles as a function of distance from the center of mass (code provided in ESA) and from the interface, as defined by the convex hull method in PUCHIK, of each assembly is shown, respectively. While the density as a function from the center of mass yields adequate results for the sphere, it fails to properly describe the density of particles that form the cylinder. The density calculated in this manner results in values greater than zero beyond the interface (*r* = 0), which is a result of the asymmetry of the shape of the assembly. At the same distance from the center of mass of the cylinder, there will be particles along the cylinder’s long axis but not along its short axis. By employing an intrinisic surface approach, PUCHIK calculates correct density values for both the sphere and the cylinder as the calculations are done, relative to the interface of the structure. As both of these structures have the same density regardless of their shape, their corresponding density plots look identical. When calculating the density, the minimum distance from the surface of the interface is determined for each particle. The center of geometry of the structure represents the maximum distance from the interface while inside the aggregate of particles. Furthermore, results from the PUCHIK software were compared to that determined by the nanoCISC algorithm for two different structures, a semispherical micelle formed by TX100 surfactants (Figure S3a), and an aspherical micelle formed by TX100 surfactants and solubilized indomethacin molecules (Figure 2a) described in our previous work.^43^ The parameter *norm_bin_count* in PUCHIK was set to 12 and nanoCISC was executed by the default parameters described in its documentation. The results of the semispherical micelle are presented in the ESI.

On the elongated nanoparticle, consisting of 6750 heavy atoms and with dimensions of ∼110 Å × 84 Å × 74 Å (Figure 2a), the nanoCISC algorithm provides inaccurate results, whereas the PUCHIK algorithm provides sensible results (Figure 2b and c). The first issue to note with the results produced with the nanoCISC algorithm is the water density (Figure 2b), where it drastically increases such that the density is larger than the expected average density value of water in bulk (∼0.033 Å^–3^). Second, the density of water calculated with the nanoCISC algorithm suggests that there is a large amount of water inside the hydrophobic core of the micelle. The results from the PUCHIK calculations, on the other hand, yield results similar to those of the spherical micelle. This shows that regardless of the shape of the nanoparticle, the densities are correctly estimated.

Due to the asymmetry of the micelle, one axis is significantly larger than the other two. Therefore, algorithms that are symmetry-dependent are prone to provide results with larger statistical error. In Figure 2b, the headgroup and tail densities reach a plateau within the core, with the headgroup density being higher than that of the hydrophobic tail, a pattern that does not resemble a stable core–shell model nanoparticle. In Figure 2c, on the other hand, the PEO density, after reaching its peak near *r* = 0, decreases to 0. This is expected as the distances are calculated relative to the convex hull of the interface, so *r* = 0 means any point on the interface. Hence, the PUCHIK method allows the core or shell thickness of an asymmetric, nonspherical nanoparticle to be calculated.

### Convex Hull vs Alpha Shape

In most cases, the convex hull provides an adequate representation of the hydrophobic core–water interface. Some inaccuracies will start to appear when the core is a bent or curved structure. For systems with features such as water-filled cavities, such as liposomes, it is preferable to model the nanoparticle interface as hollow.

The convex hull is not able to correctly describe such structures by definition, as it will enclose every bend and gap. A better approach to this problem is the use of alpha shapes, which can be customized to model such nanoparticles perfectly.

**Figure 3:**
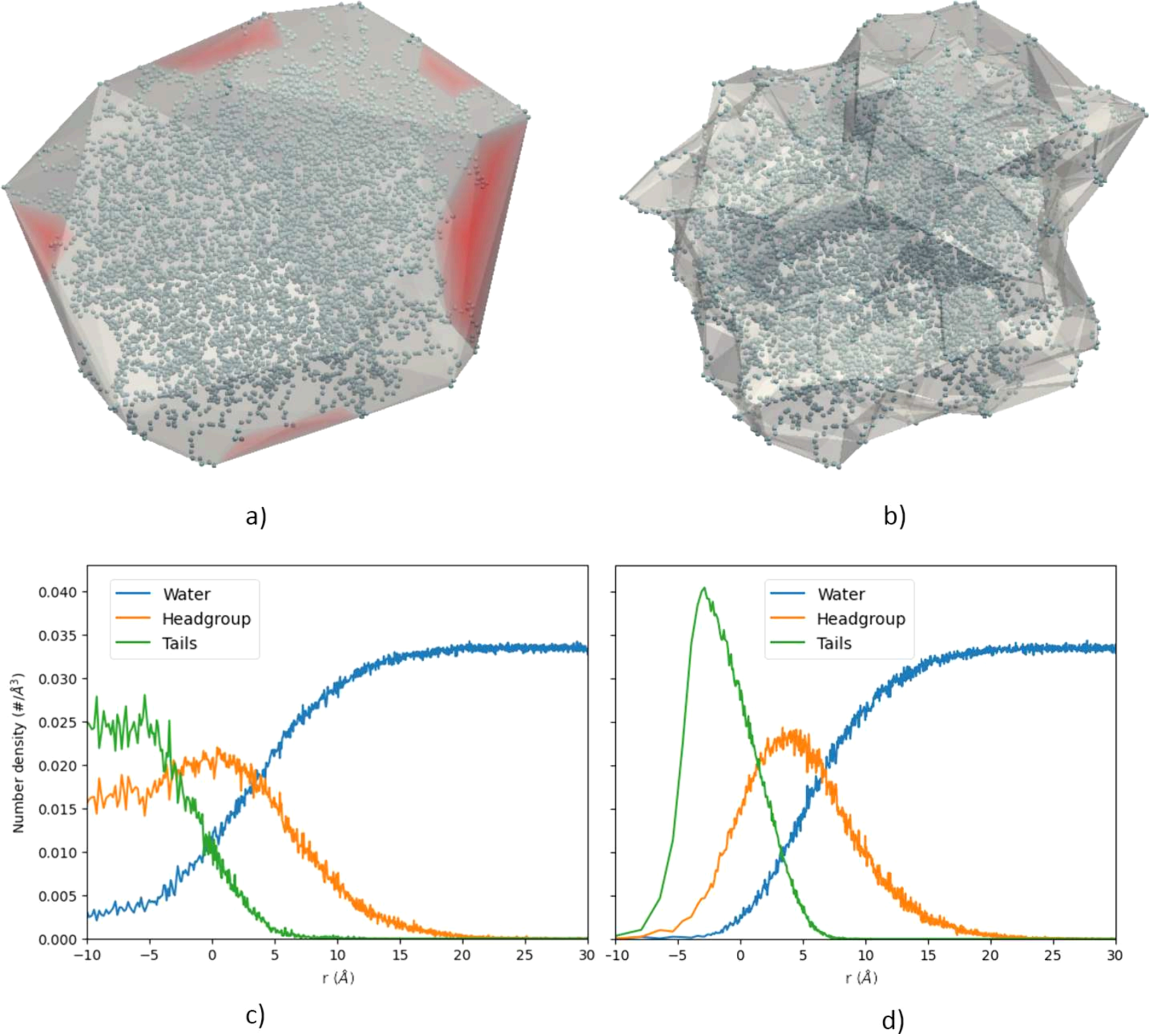
Same nanoparticle modeled using (a) a convex hull and (b) an alpha shape. Number of atoms per volume of water, hydrophobic tail, and hydrophilic head calculated using (c) a convex hull and (d) an alpha shape. *r* is the distance of the particle from the convex hull/alpha shape.

Figures 3a and 3b show a comparison of a surfactant-based nanoparticleś interface using both methods. Figure 3a shows the convex hull of a nanoparticle. Although the red areas are part of the hull, they contain almost no atoms belonging to the nanoparticle and are filled with water molecules. Therefore, the density calculations might show a higher density of water inside the core of the nanoparticle, which is the case in Figure 3c. However, the alpha shape (Figure 3b) forms a concave interface, better fitting to the overall shape of the nanoparticle. Figure 3d shows a significantly smaller water density inside of the nanoparticle when the alpha shape is used compared to that using the convex hull. Moreover, since the alpha shape typically encloses a smaller volume than its convex hull counterpart, while containing the same number of points, the number density (atoms per volume) is higher using an alpha shape than using a convex hull. Finally, it is important to note that calculating the alpha shape is computationally more expensive than the convex hull, representing a tradeoff between performance and precision. A comparison can be found in Figure S6 in the ESI.

### Performance

The performance of PUCHIK throughout the development was improved by first identifying the bottleneck functions of the code. These functions are separated from the code and compiled by using Cython. The ESI provides details on how the profiling and compilation were conducted. This gives a slight performance boost of ∼6%. Finally, the code is parallelized to use multiple CPU processes simultaneously. The results are shown in Table 1. The system used for this benchmark contained ∼51 000 water molecules and ∼1100 interface atoms. Hydrogen atoms were excluded from the computation. Implementing a simple parallelization through multiprocessing increases the performance of the noncompiled code dramatically. The rate of change in computational time, depending on the number of frames, is ∼200% less for parallelized code than for the single process version. Furthermore, the parallelization of a compiled code offers an additional ∼11% performance boost.

**Table 1:** Execution Time Per Frame for Different Techniques to Compute Densities in the PUCHIK Algorithm

optimization technique	execution time (seconds per frame)
Python SP	0.40
Python + Cython SP	0.37
Python MP	0.13
Python + Cython MP	0.12

**Figure 4:**
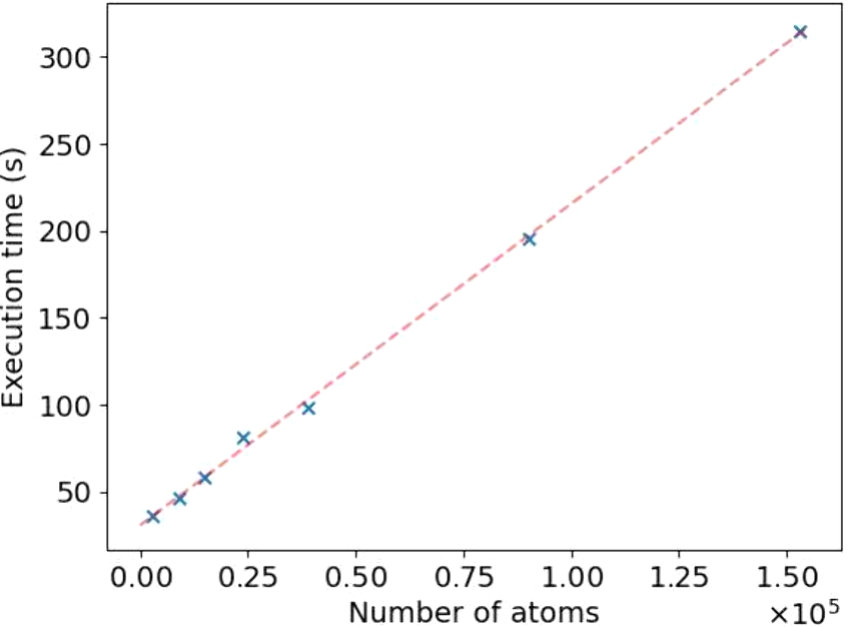
Linear dependence of the execution time on the number of particles.

Finally, the time complexity of the code can be estimated as *O*(*mN*), where *N* is the number of particles for density calculations and *m* is the number of vertices on the convex hull. With a constant *m*, the execution time increases linearly with an increasing *N* (Figure 4). These calculations were performed using a trajectory of 168 989 atoms.

## Conclusions

This work introduces PUCHIK, a Python package specifically designed for the quantitative characterization of nanoparticle interfaces from MD trajectories. PUCHIK addresses the limitations of the current tools by providing a robust framework for analyzing both spherical and aspherical interfaces. The modular nature of PUCHIK makes it easy for the users to add functionality to the code, so that further analysis can be done using the definition of the location of the interface of the hydrophobic core of the nanoparticle. The results provided by our code enable the analysis of important quantities, enabling a deeper understanding of the structure–property relationships present in these systems.

Furthermore, the limitations of using a convex hull are discussed. It was shown that, although convex hull provides a good approximation for spherical and elongated ellipsoids, interfaces of structures that contain bends, curves, and cavities will not be described properly as a convex hull. The convex hull will encompass the grooves and gaps leading to an oversimplified representation of these complex interfaces. To address this issue, another implementation for interface construction was presented. This implementation utilizes alpha shapes to produce a more fitting interface for a nanoparticle of an arbitrary shape. By having the ability to use both algorithms, PUCHIK allows users to analyze nanoparticles of any shape with one tool.

Finally, the manuscript also provides guidelines to make computationally intensive codes dramatically faster by utilizing one or a combination of different methods, such as compilation, multiprocessing, and simple algorithmic optimizations. Additionally, simply refactoring the code to enable parallel execution can dramatically improve performance, with speedups scaling according to the number of CPU cores employed. Using these optimization strategies, PUCHIK has improved performance in comparison to the Pytim software^44^ (see the ESI), which has some of the same functionality.

PUCHIK will play an integral role in linking the molecular-scale detail available from molecular dynamics (MD) simulations to the observations made experimentally when studying nanoparticles. This tool will make it that much easier for users to utilize the results of their simulations to inform the modeling of experimental data. For example, the ability to accurately define the shape that is formed by the core and the corona of the nanoparticle is useful, because that information is necessary in order to choose appropriate models for understanding the data from neutron scattering experiments, which are a nice complement to MD simulations when studying nanoparticles. Additionally, being able to measure the type and amount of the different atomic species found in the core (and corona) of a nanoparticle is useful when assessing the scattering length density, which is used in the modeling of neutron scattering data of such systems. Therefore, in addition to providing the molecular-scale detail that is not assessable experimentally, this tool will provide further information to assist in the modeling of the experimental data gathered on these increasingly complex systems.

## Data Availability

The PUCHIK software package and the input files for the simulations reported in this manuscript are available for download from the Web site: https://github.com/hrachishkhanyan/PUCHIK/tree/alpha_shapes.

## Supporting Information Available

(a) Instructions for installing PUCHIK; (b) an example of the code required to utilize PUCHIK; (c) a brief discussion of the algorithm used within PUCHIK; (d) a description of the results from the density calculation within PUCHIK; (e) discussion of code optimization; (f) comparison of intrinsic densities measured with nanoCISC^46^ and PUCHIK; (g) a description of the profiling protocol used in PUCHIK; (h) a comparison of the performance of PUCHIK using a convex hull and alpha shapes for interface construction; and (i) a comparison of the performance of PUCHIK and Pytim^44^ (PDF)
