# Supplementary material for: PUCHIK: A Python Package To Analyze Molecular Dynamics Simulations of Aspherical Nanoparticles
Source: J Chem Inf Model. 2025 Feb 10;65(4):1694–701. doi: 10.1021/acs.jcim.4c02128 (PMC11863366; doi:10.1021/acs.jcim.4c02128)
Supplement: Supplementary file 1 — ci4c02128_si_001.pdf [file ci4c02128_si_001.pdf]

Supporting information for "PUCHIK: A Python package to analyze molecular dynamics simulations of aspherical nanoparticles"

Hrachya Ishkhanyan,[*,†,‡] Alejandro Santana-Bonilla,[‡] and Christian D. Lorenz[*,¶]

†*Institute for Informatics and Automation Problems of the National Academy of Sciences of the Republic of Armenia, Yerevan, Republic of Armenia*

‡*Department of Physics, King's College London, London, WC2R 2LS, United Kingdom*

¶*Department of Engineering, King's College London, London, WC2R 2LS, United Kingdom*

E-mail: hrachishkhanyan@gmail.com; chris.lorenz@kcl.ac.uk

Usage

PUCHIK can be installed directly from the Python Package Index (PyPI) using pip:

```
pip install PUCHIK
```

Pytest[1] can be used to run the tests. It can be done simply by executing *pytest* from the package directory in the terminal:

```
pytest
```

A basic calculation of density can be executed using test structures included in PUCHIK:

```
m = Interface('<PATH_TO_PUCHIK>\PUCHIK\test\test_structures\InP_cylinder.pdb')
m.select_atoms('all')
```

```
m.select_structure('resname UNL')
dist, dens = m.calculate_density('all', norm_bin_count=8)
```

The resulting number density from distance can be plotted using the *matplotlib* package:

```
plt.plot(dist, dens)
plt.ylabel('Number density (#/$\AA^3$)')
plt.xlabel('Distance ($\AA$)')
plt.show()
```

PUCHIK Algorithm

The workflow of PUCHIK program is presented in S1. After providing topology and trajectory details, PUCHIK requires the selection of atoms composing the desired structure (nanoparticle, shell, core, etc.). An observable can then be calculated. Currently, PUCHIK supports the calculation of three parameters: number density, volume and the number solubilized molecules. Each of the methods responsible for the calculation call the **create_hull** method. This method creates the interface (hull or alpha shape) for the atom selection. The resulting interface is stored within an **Interface** object, ensuring that it is calculated only once and reused for subsequent observable calculations. This modular approach simplifies the addition of new parameters in future versions of PUCHIK.

Details on the Results

The number density of particles as a function of distance from the center of mass was calculated using this code:

```
import MDAnalysis as mda


def calculate_radial_density(u: mda.Universe, selection: str, ref: str, layer_count=15):
    pbc = u.dimensions[0]
```

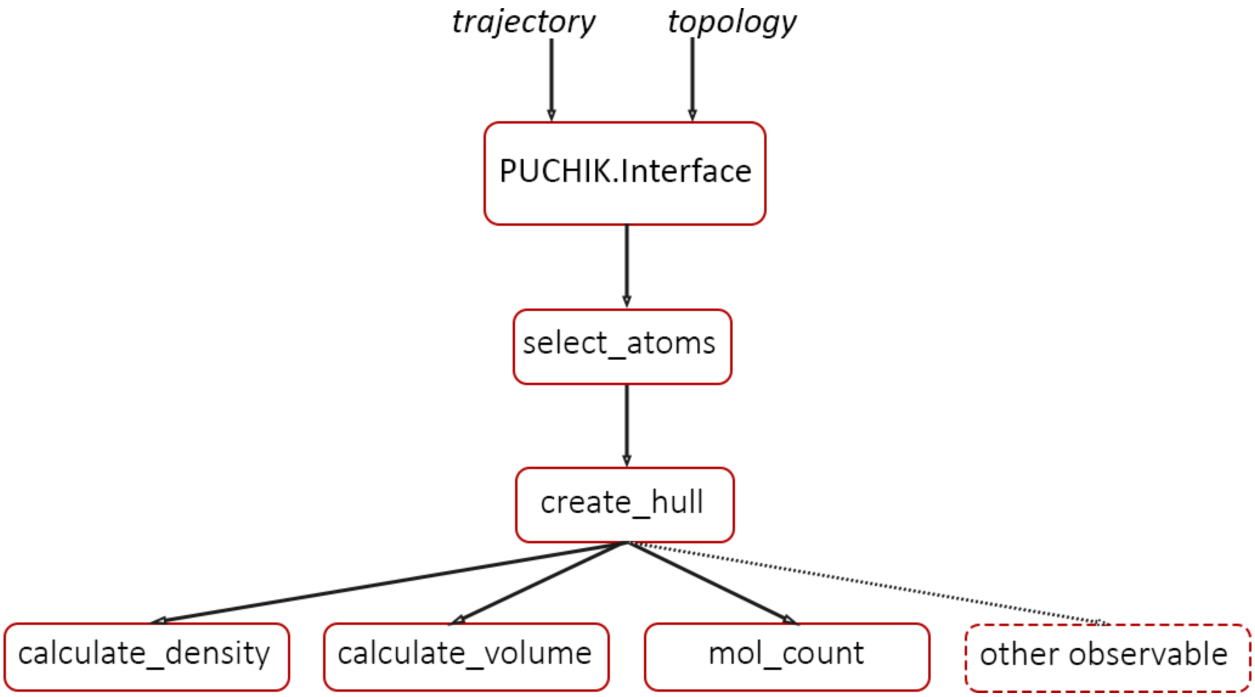

Figure S1: Schematic diagram describing the workflow of the algorithm. The hull can be created separately and is independent of the calculations of the observables.

```
density_ag = u.select_atoms(selection)


densities = np.zeros(layer_count)

distances = np.zeros(layer_count)


ls, step = np.linspace(0, pbc, layer_count, retstep=True)


for index, i in enumerate(ls):

    layer_volume = 3 / 4 * np.pi * ((i + step) ** 3 - i ** 3)

    n_layer_atoms = density_ag.select_atoms(f'sphlayer {i} {i + step} ( {ref} )').n_

    densities[index] = (n_layer_atoms / layer_volume)

    distances[index] = i


return distances, densities
```

The code divides the simulation box into layers, whose thickness are determined by the number of layers and the size of the simulation box. The density is then calculated for each layer by determining the number of atoms inside of the layer and dividing it by the volume of the layer.

Strategies for Optimizing Code Performance

Algorithm implementation and further optimizations are crucial steps for enhancing performance, especially when aiming to scale to extended systems. There are simple ways to enhance ones code performance without significant computer science knowledge and experience. This work introduces accessible code optimization techniques implemented in Python, demonstrating how to achieve significant performance enhancements with minimal computer science background. These concepts are broadly applicable and can inspire efficient implementations across various other projects facing similar challenges. Efficient code optimization relies on identifying performance bottlenecks. Parallelization of an existing code is one way to ensure that it utilizes the available computational power as efficiently as possible, making the execution dramatically faster. However, it requires the existing code to be structured in a way that allows for independent tasks to be executed in independently. The calculations of the hull and density are done separately for each frame making PUCHIK ideal to run in parallel. The *multiprocessing* module from the Python standard library is used in this package to parallelize these functions. The *cpu_count* parameter allows the user to set the amount of logical processors they want to utilize for the calculation. The default value is set

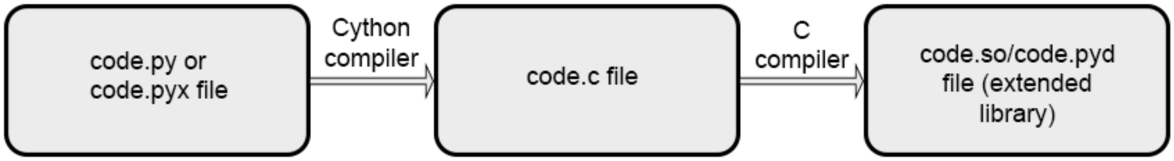

Figure S2: Schematic diagram providing a successful Cython workflow.

to the maximum number of logical processors available on the user's computer.

The standard implementation of Python (CPython) is in most part an interpreted language. However, several techniques and frameworks can be utilized to compile Python code to gain the performance advantage of a compiled language. In PUCHIK, Cython was used to compile computationally intensive chunks of the code.

While algorithmic optimizations are beyond the scope of this work, there are some easy-to-implement techniques that can enhance performance further. One such technique used in PUCHIK is memoization. For each frame of the trajectory, the convex hull is cached, allowing it to be quickly reused for other calculations, such as volume or solubilization.

Code optimization by compilation

In its standard implementation (CPython), Python is an interpreted language, meaning that the code does not need to be compiled into an executable before being run. There is, however, an intermediate compilation step performed (into bytecode), which is subsequently executed by the interpreter rather than processed as a machine code. That is a significant optimization, but not enough to compete with some compiled languages. However, there are different frameworks and Python implementations available, that allow Python code to be compiled. Examples are Cython, Numba,[2] Pypy,[3] etc. In this case and following the structure of the Python project, Cython has been selected to provide further performance optimization. In simple words, Cython is a superset of Python, that can compile a Python (.py file), or an extended Python (.pyx file) script into a C code. The resulting C code is then compiled into an extension module, which is imported and used in pure Python code (Fig. S2). Any Python file can be compiled, but Cython offers additional features, such as type definitions, global interpreter lock (GIL) release, and working directly with C extensions, to name a few, that can further improve performance. However, it is worth mentioning that this method will not universally offer a significant difference in performance. If the developer is already using

C extension libraries such as NumPy, SciPy, for computationally intensive tasks, there might be a little to no performance gain. On the other hand, Python itself is continuously evolving, with significant improvements in speed and efficiency. These advancements are reducing the need for a separate compilation step in many scenarios as will be demonstrated in the next section. In PUCHIK, Cython was used to compile several bottleneck functions, such as the distance calculation function, as part of the code optimization, effectively increasing the performance of the calculation. Namely, compiled functions are **find_distance** and the utility functions used by it: **_is_inside**, **point_in_hull**, **point_in_alpha_shape** and **_point_in_tetrahedron**. These are used to determine whether a point is inside of the hull or alpha shape. Pure Python and Cython versions can be found in the PUCHIK's repository under the following paths - **PUCHIK/grid_project/core/utils_python.py** and **PUCHIK/grid_project/core/utils.pyx**, respectively.

Comparison with other algorithms

Figures S3b & S3c show the results when using the nanoCISC and PUCHIK algorithms, respectively, to analyze the semi-spherical micelle shown in Figure S3a. Both algorithms yield similar density values, with the main differences being slight shifts in the plots, which is expected due to the differences in interface construction metods. The nanoCISC algorithm describes the interface employing user-defined anchor points, after which the interface is constructed by interpolating between these points, whereas the PUCHIK code uses any number of given atoms to construct a convex hull around them.

In terms of performance, PUCHIK was benchmarked against Pytim,[4] a well-established Python package for interface construction and analysis. The generalized ITIM algorithm in Pytim also utilizes alpha-shapes in order to construct interfaces. Performance comparison has been conducted on a GROMACS trajectory consisting of 169,620 particles and 600 frames. During testing, the nanoparticle interface was constructed using both PUCHIK and

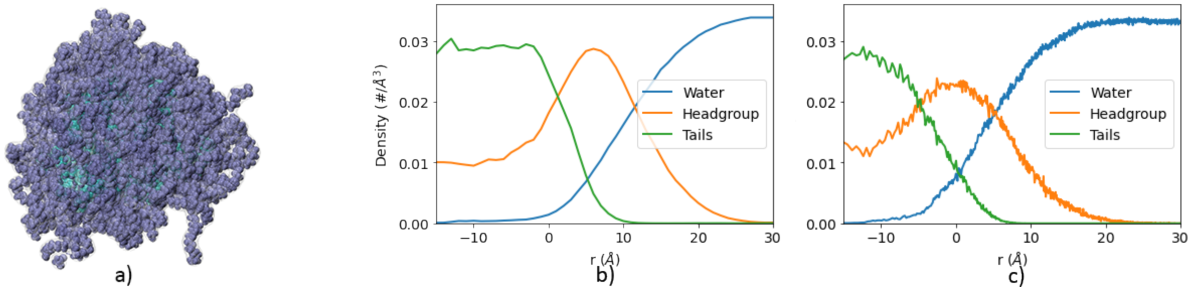

Figure S3: a) Snapshot of a semi-spherical TX100 micelle. Density profiles of water, hydrophobic tail and hydrophilic head of Triton X-100 surfactant. Comparison of results from two algorithms - b) nanoCISC and c) PUCHIK.

GITIM, and the water density profile was calculated. The computation was repeated with subsets of 10, 20, 60, 100, and 600 frames, and the execution times were recorded. The results, presented in Figure S4, demonstrate that while both algorithms perform efficiently, PUCHIK shows a performance advantage, indicated by a smaller slope in its linear execution time dependence on the number of frames processed.

Profiling

This work presents a simple profiling protocol, utilizing the cProfile library in CPython, to pinpoint computationally intensive areas within a software package. cProfile provides comprehensive statistics showing the performance and execution time of every single function call. To improve the readability of the results from cProfile, gprof2dot[5] and Graphviz[6] were used to visualize these statistics.

PUCHIK was profiled following this protocol and the output is visualized on Figure S5. The percentage value displayed on each node of the graph indicates the proportion of the total execution time that is attributed to that particular function. The *_calc_dens* is the main function responsible for the computation of density and takes approximately 85.46% of the computational time (Fig. S5a). This function itself calls several other functions, with the *_create_hull* function takes more than half of that time to execute. The reason behind this is *make_coordinates* utility function, which transforms a three dimensional grid

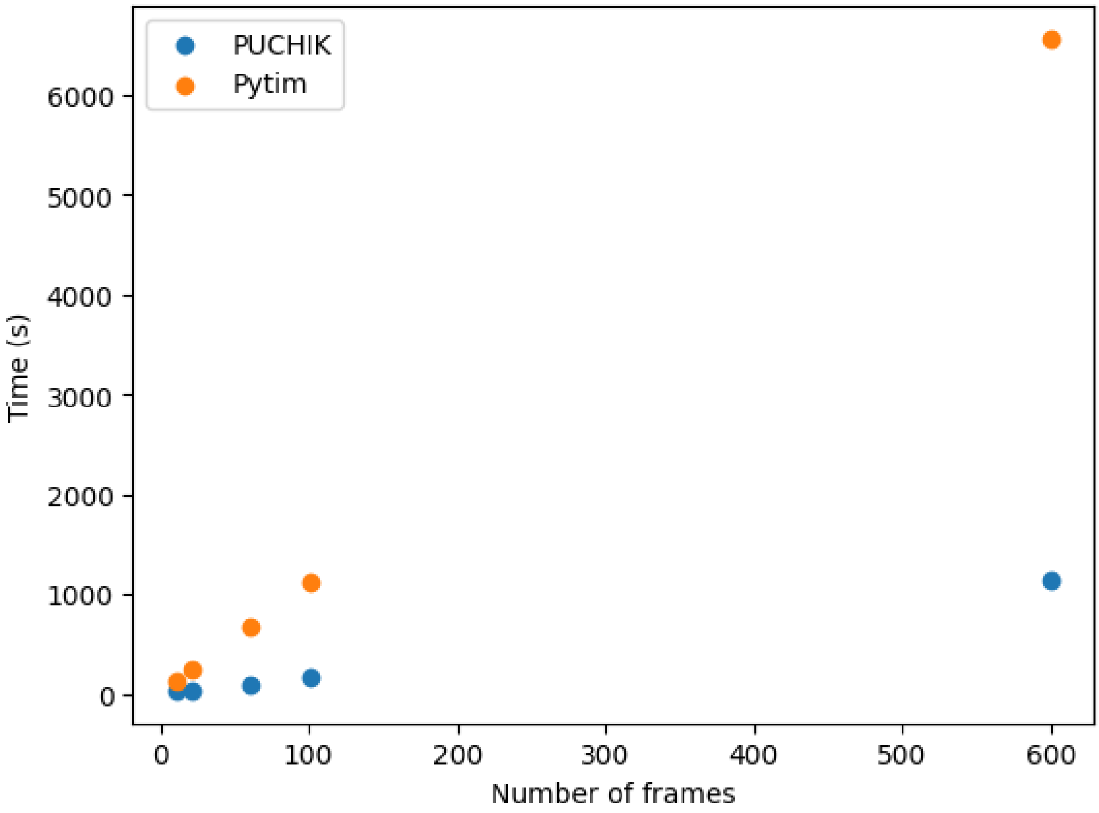

Figure S4: Performance comparison between the Pytim and PUCHIK packages.

of points into coordinates. A simple optimization of this function dramatically reduces its execution time, hence the overall execution time of the PUCHIK package (Fig. S5b). In this code, after this optimization, there is not much to be done in pure Python. The two functions (*ray_inside_test* and *signed_distance*) use a C++ library in the background and their execution is already fast. On the other hand, the *_calc_mesh* method can be further improved by utilizing code compilation.

Additional notes

PUCHIK currently has the following versions for its dependencies:

```
Cython==3.0.10

numpy==1.26.0

scipy==1.14.0
```

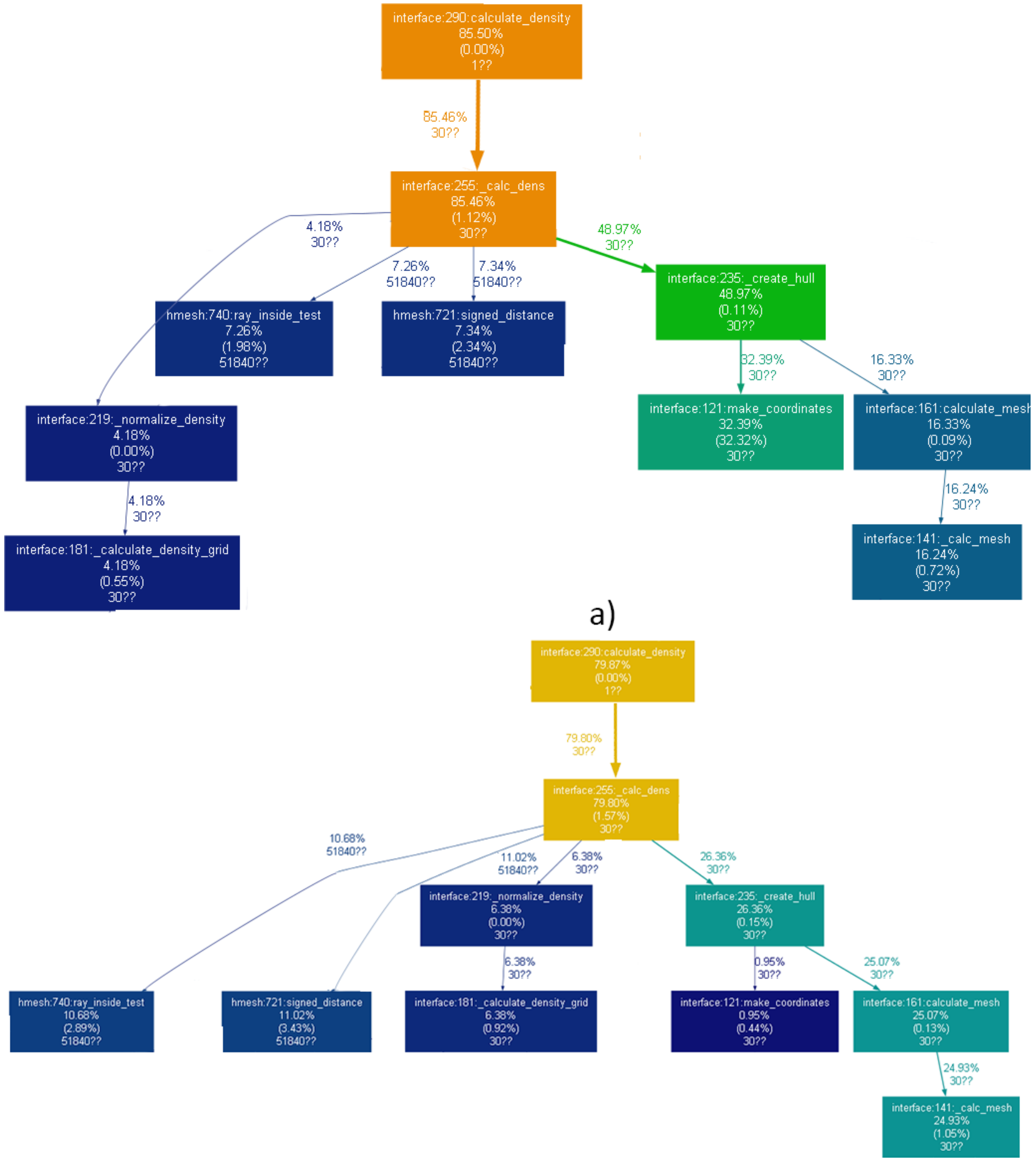

Figure S5: A visualization of a part of the profiling. In a) a computationally intensive function, *make_coordinates* was identified and optimized boosting the overall performance (b).

```
MDAnalysis==2.8.0

pygel3d==0.5.2
```

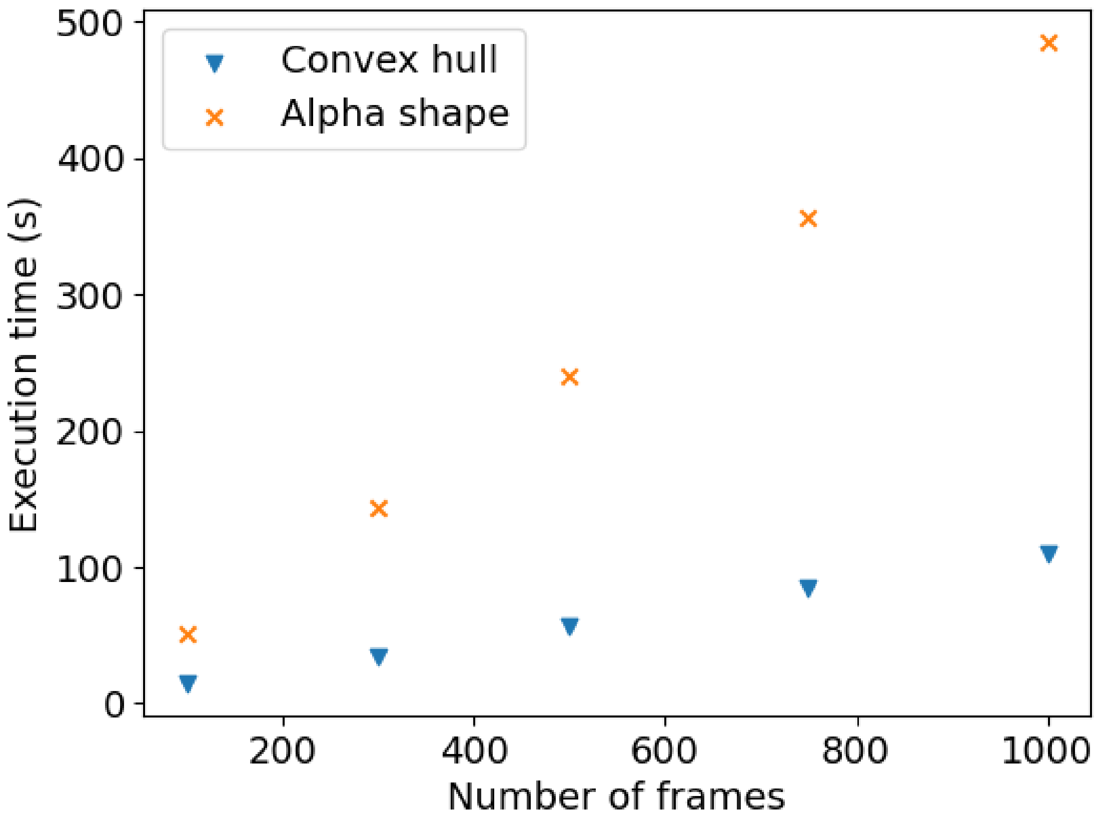

Figure S6: Performance comparison of PUCHIK using convex hull vs alpha shape for interface construction.

The benchmarking was done on a machine running an AMD Ryzen 5 3550H CPU (8 logical cores) with 16gb of DDR4 RAM.

References

(1) Krekel, H.; Oliveira, B.; Pfannschmidt, R.; Bruynooghe, F.; Laugher, B.; Bruhin, F. pytest x.y. 2004; `https://github.com/pytest-dev/pytest`.

(2) Lam, S. K.; Pitrou, A.; Seibert, S. Numba: A LLVM-based Python JIT Compiler. *Proceedings of LLVM-HPC 2015: 2nd Workshop on the LLVM Compiler Infrastructure in HPC - Held in conjunction with SC 2015: The International Conference for High Performance Computing, Networking, Storage and Analysis* **2015**, *2015-January*.

(3) https://doc.pypy.org/en/latest/index.html.

(4) Sega, M.; Handal, G.; Fábián, B.; Jedlovszky, P. Pytim: A Python Package for the Interfacial Analysis of Molecular Smulations. *J. Comput. Chem.* **2018**, *39*, 2118–2125.

(5) https://github.com/jrfonseca/.

(6) Gansner, E. R.; North, S. C. An open graph visualization system and its applications to software engineering. *Software: Practice and Experience* **2000**, *30*, 1203–1233.
